# Bone Mineral Density and All-Cause Mortality in Patients with Nondialysis Chronic Kidney Disease: Results from KNOW-CKD Study

**DOI:** 10.3390/jcm12051850

**Published:** 2023-02-25

**Authors:** Sang Heon Suh, Tae Ryom Oh, Hong Sang Choi, Eun Mi Yang, Chang Seong Kim, Eun Hui Bae, Seong Kwon Ma, Kook-Hwan Oh, Young Youl Hyun, Suah Sung, Soo Wan Kim

**Affiliations:** 1 Department of Internal Medicine, Chonnam National University Medical School, Gwangju 61469, Republic of Korea; 2 Department of Internal Medicine, Chonnam National University Hospital, Gwangju 61469, Republic of Korea; 3 Department of Pediatrics, Chonnam National University Medical School, Gwangju 61469, Republic of Korea; 4 Department of Pediatrics, Chonnam National University Hospital, Gwangju 61469, Republic of Korea; 5 Department of Internal Medicine, Seoul National University Hospital, Seoul 03080, Republic of Korea; 6 Department of Internal Medicine, Kangbuk Samsung Hospital, Sungkyunkwan University School of Medicine, Seoul 03181, Republic of Korea; 7 Department of Internal Medicine, Eulji Medical Center, Eulji University, Seoul 01830, Republic of Korea

**Keywords:** bone mineral density, chronic kidney disease, cohort, mortality, osteoporosis

## Abstract

Despite the clear association between low BMD and all-cause mortality in the general population, the association has not been validated in patients with nondialysis CKD. To investigate the association of low BMD with all-cause mortality in this population, a total of 2089 patients with nondialysis CKD at stages 1 to predialysis 5 were categorized into normal BMD (T-score ≥ −1.0), osteopenia (−2.5 < T-score < −1.0), and osteoporosis (T-score ≤ − 2.5) by the BMD at femoral neck. The study outcome was all-cause mortality. Kaplan–Meier curve depicted a significantly increased number of all-cause death events in the subjects with osteopenia or osteoporosis during the follow-up period compared with subjects with normal BMD. Cox regression models demonstrated that osteoporosis, but not osteopenia, was significantly associated with an increased risk of all-cause mortality (adjusted hazard ratio 2.963, 95% confidence interval 1.655 to 5.307). Smoothing curve fitting model visualized a clear inverse correlation between BMD T-score and the risk of all-cause mortality. Even after recategorizing the subjects by BMD T-scores at total hip or lumbar spine, the result was similar to the primary analyses. Subgroup analyses revealed that the association was not significantly modified by clinical contexts, such as age, gender, body mass index, estimated glomerular filtration rate, and albuminuria. In conclusion, low BMD is associated with an increased risk of all-cause mortality in patients with nondialysis CKD. This emphasizes that the routine measurement of BMD by DXA may confer an additional benefit beyond the prediction of fracture risk in this population.

## 1. Introduction

Bone mineral density (BMD) measured by dual-energy X-ray absorptiometry (DXA) is commonly used to predict the risk of fracture in the general population [1], though its use has long been limited among patients with chronic kidney disease (CKD) [2]. CKD mineral and bone disorder (CKD-MBD) develops as CKD progresses, which is a complex disorder in bone and mineral metabolism among patients with CKD [3]. Based on the concern that BMD measured by DXA might not precisely reflect bone quality in patients with CKD, as the subtypes of CKD-BMD can affect BMD scores [4], the routine measurement of BMD by DXA was not recommended by the 2009 Kidney Disease: Improving Global Outcomes (KDIGO) guideline for CKD-MBD [2]. Yet, BMD tends to be low in patients with CKD compared with the general population and continues to decrease as CKD progresses [5,6], while the risk of fracture is also higher in patients with CKD than in the general population [4]. Indeed, mounting evidence from observational studies suggests that low BMD is associated with an increased risk of fracture both in nondialysis [7] and dialysis-dependent [8] CKD patients. Thus, the 2017 KDIGO guideline for CKD-MBD currently recommends the use of BMD to assess the risk of fracture in patients with CKD [9].

Beyond the prediction of fracture risk, low BMD is also associated with vascular calcification, cardiovascular disease (CVD), and all-cause mortality in the general population [10,11,12,13,14]. Likewise, a recent cohort study of patients with nondialysis CKD revealed that low BMD is associated with the progression of coronary artery calcification and adverse cardiovascular outcomes [15]. Another study from the KNOW-CKD cohort recently reported that low BMD is associated with the risk of incident end-stage renal disease (ESRD) among patients with nondialysis CKD [16], in whom both osteopenia (adjusted HR 1.14, 95% confidence interval (CI) 0.92 to 1.41) and osteoporosis (adjusted HR 1.43, 95% CI 1.01 to 2.04) were significantly associated with the risk of incident ESRD. In contrast, despite the clear association between low BMD and all-cause mortality in the general population, the association has been validated only in patients with ESRD [17,18,19,20] but not in patients with nondialysis CKD.

Therefore, we aimed to investigate the association of low BMD with all-cause mortality in patients with nondialysis CKD. To test the hypothesis that low BMD is associated with an increased risk of all-cause mortality in this population, we took advantage of BMD T-scores at femur neck, total hip, and lumbar spine to define osteopenia and osteoporosis and evaluated the risk of all-cause mortality related to osteopenia and osteoporosis in a cohort of 2098 patients with nondialysis CKD.

## 2. Materials and Methods

### 2.1. Study Design

**Figure 1:**
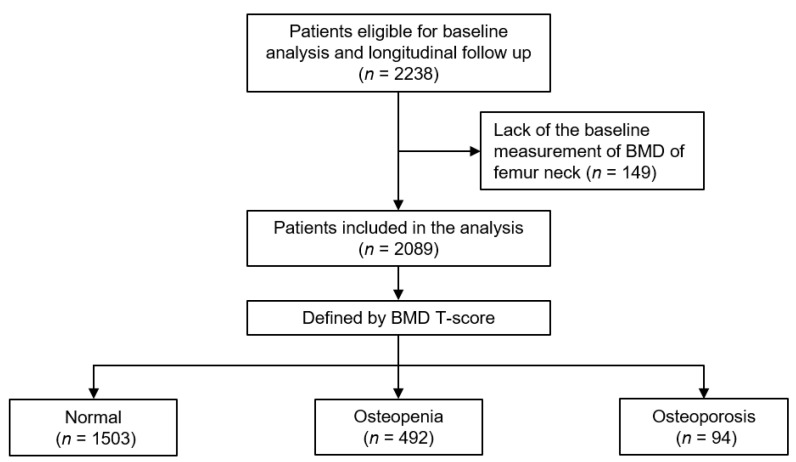
Flow diagram of the study participants. Abbreviations: BMD, bone mineral density.

The Korean Cohort Study for Outcomes in Patients with Chronic Kidney Disease (KNOW-CKD) has been previously described (NCT01630486 at http://www.clinicaltrials.gov accessed on 18 January 2023) [21]. The study was conducted in accordance with the principles of the Declaration of Helsinki. The study protocol was endorsed by the institutional review board at each participating center. Patients with CKD at stages 1 to predialysis 5 and with ages 20 to 75 years were enrolled from 2011 through 2016. All participants agreed to submit informed consent. For the close observation of the medical condition, participants were allowed to visit the nephrology clinic upon the recognition of physical abnormalities, and each participating center recorded study outcomes. After excluding those without baseline measurement of BMD at femur neck (*n* = 149), a total of 2089 patients were finally included and analyzed (Figure 1). The study observation period ended on 31 March 2022. The median follow-up duration was 7.966 years.

### 2.2. Data Collection from Participants

Demographic information included age, gender, Charlson comorbidity index, primary renal disease, smoking history, and medication history (angiotensin-converting enzyme inhibitors and angiotensin II receptor blockers (ACEIs/ARBs), diuretics, number of anti-HTN drugs, statins). The height and weight of study participants were measured by trained staff members. Body mass index (BMI) was calculated as weight divided by height squared. Systolic and diastolic blood pressures (SBP and DBP) were measured by an electronic sphygmomanometer after seated rest for 5 min. Venous samples were collected following overnight fasting to determine hemoglobin, albumin, total cholesterol, low-density lipoprotein cholesterol (LDL-C), high-density lipoprotein cholesterol (HDL-C), triglycerides (TG), fasting glucose, 25-hydroxyvitamin D (25(OH)D), total calcium, phosphorus, and intact parathyroid hormone (iPTH), high-sensitivity C-reactive protein (hs-CRP), and creatinine (Cr) levels at the baseline. Chronic Kidney Disease Epidemiology Collaboration equation using serum Cr level was adopted to calculate estimated glomerular filtration rate (eGFR) [22]. CKD stages were determined by the Kidney Disease Improving Global Outcomes guidelines [23]. Urine albumin-to-Cr ratio (ACR) was measured in random, preferably second-voided, spot urine samples. To collect echocardiographic data, complete two-dimensional M-mode and Doppler studies were performed via standard approaches by cardiologists at the participating hospitals who were blinded to the clinical data. M-mode examination was performed according to American Society of Echocardiography guidelines [24]. LV mass index (LVMI) was calculated by normalizing LV mass to height^2^ (g/m^2^) [24].

### 2.3. Measurement of BMD

BMD was assessed by a DXA system (Hologic, Marlborough, MA, USA). BMD was measured at femur neck, total hip, and lumbar spine (L1–L4). Results were expressed as T-score (standard deviation relative to 20- to 30-year-old Koreans according to the Korean Nutrition Health and Nutrition Examination Survey reference).

### 2.4. Exposure and Study Outcomes

According to WHO classification, the subjects were categorized into normal BMD (T-score ≥ −1.0), osteopenia (−1.0 > T-score > −2.5), and osteoporosis (T-score ≤ −2.5) by the BMD at femur neck (Figure 1). The study outcome was all-cause mortality. For accuracy of the clinical outcomes, the participating investigators cross-checked all outcome events by regularly visiting the other participating medical centers to review the case report forms.

### 2.5. Statistical Analysis

To compare the baseline characteristics by femur neck BMD, one-way analysis of variance and χ^2^ test were used for continuous and categorical variates, respectively. Values for categorical variables were given as numbers (percentage), while values for continuous variables were given as means ± standard deviations or, if standard deviation was high, were given as median [interquartile range]. Cumulative incidences of outcome events were visualized using Kaplan–Meier curves and were analyzed by log-rank test. Participants with any missing data were excluded from further analyses. To evaluate the association between low BMD and the risk of all-cause mortality, Cox proportional hazard regression models were adopted. The study visits were scheduled at baseline, 6 months, 1 year, and annually thereafter. The participants who did not appear at the regular follow-up visit were censored unless specific outcome events, including all-cause death and initiation of renal replacement therapy (dialysis and transplantation), etc., were not recorded. The cause of death events in the KNOW-CKD study was categorized as the following: cardiovascular, infection, malignancy, sudden cardiac death, liver disease, accident, suicide, others, unknown. The overall incidence of all-cause death events was 8.4% (175 out of 2086). Patients lost to follow-up were censored at the date of the last visit. Models were constructed after adjusting for confounding variables, which were all recorded at baseline. The number of participants who remained in adjustment model 4 was 1333, 422, and 831 for normal, osteopenia, and osteoporosis groups by femur neck BMD, respectively. The results of Cox proportional hazard models were presented as HRs and 95% CIs. Smoothing curve fitting (penalized spline method) was used to assess the relationship between BMD T-scores (as a continuous variable) with the risk of all-cause mortality, where the hazard ratio at the nadir was designated as 1 and was proportionally calculated for the other points. To confirm our findings, we performed sensitivity analyses. First, we evaluated the association of low BMD at total hip and lumbar spine, on top of BMD status at femur neck, with risk of all-cause mortality. Second, we excluded the subjects at CKD stage 1 because their kidney function was too close to normal to represent CKD population well. Third, we excluded subjects at CKD stage 5 because the subjects were relatively small in number and may have exaggerated the association between low BMD and the risk of all-cause mortality due to far advanced CKD. Fourth, we repeated Cox regression analysis by using multiple imputation by random sampling method. Fifth, we included total calcium, phosphorus, and iPTH in the regression models as covariables. To test whether the association between low BMD and all-cause mortality is modified by clinical factors, we planned prespecified subgroup analyses. Two-sided *p*-values of < 0.05 were considered statistically significant. Statistical analysis was performed using SPSS for Windows version 22.0 (IBM Corp., Armonk, NY, USA) and R (version 4.1.1; R project for Statistical Computing, Vienna, Austria).

## 3. Results

### 3.1. Baseline Characteristics

To describe the baseline characteristics, study participants were divided into three groups by BMD T-scores at femur neck: normal, osteopenia, and osteoporosis (Table 1). The mean follow-up duration significantly differed by femur neck BMD, as it was longest in subjects with normal BMD and was shorted in subjects with osteoporosis. The mean age was lowest in subjects with normal BMD and was highest in subjects with osteoporosis. The proportion of subjects with a Charlson comorbidity index of ≥3 and diabetes mellitus was significantly higher in subjects with osteoporosis. The proportion of current smokers was significantly higher in subjects with normal BMD. The frequencies of diuretic use, medication of three or more antihypertensive drugs, and statin use were significantly higher in subjects with osteoporosis. BMI, waist circumference, and DBP were significantly higher in subjects with normal BMD. Hemoglobin, albumin, total cholesterol, LDL-C, and TG levels were also significantly higher in subjects with normal BMD, whereas mean fasting glucose level was highest in subjects with osteoporosis. Serum total calcium was significantly higher in subjects with normal BMD, while serum phosphorus and iPTH levels were highest among subjects with osteoporosis. Compared with subjects with normal BMD, subjects with osteopenia or osteoporosis were related to heavier albuminuria and lower eGFR. The echocardiographic findings of study participants also significantly differed by femur neck BMD (Supplemental Table S1), as LVMI and E/e’ were significantly increased in subjects with osteopenia or osteoporosis, whereas LVEF was best preserved in subjects with normal BMD, although LV end-diastolic and end-systolic dimensions were significantly decreased in subjects with osteopenia or osteoporosis. The frequency of valve calcification was also significantly higher in subjects with osteopenia or osteoporosis. To summarize, subjects with low BMD were related to a substantial burden of comorbidities.

### 3.2. Association of Low BMD with All-Cause Mortality in Patients with CKD

**Figure 2:**
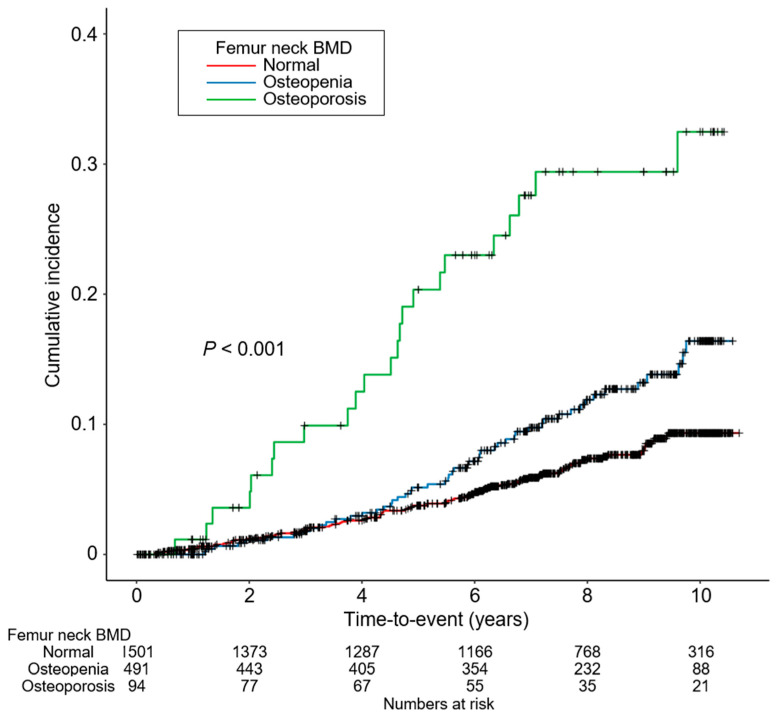
Kaplan–Meier survival curve for cumulative incidence of all-cause mortality by femur neck BMD. *p*-value by log-rank test. Abbreviations: BMD, bone mineral density.

**Figure 3:**
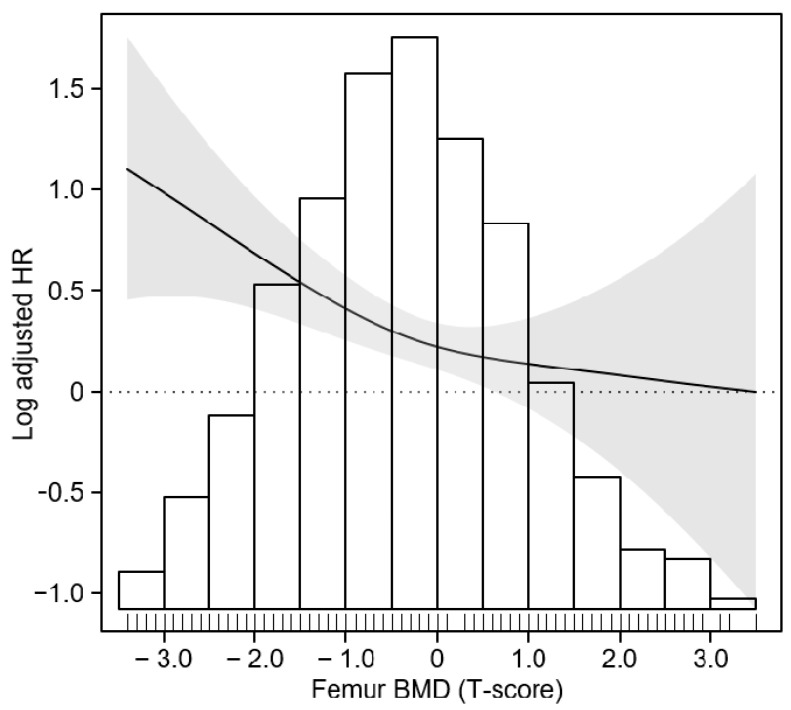
Restricted cubic spline of femur neck BMD on all-cause mortality. Adjusted HR of non-HDL-C as a continuous variable for composite CV event is depicted. The model was adjusted for age, sex, Charlson comorbidity index, primary renal disease, smoking status, medication (ACEIs/ARBs, diuretics, number of anti-HTN drugs, statins), BMI, SBP, hemoglobin, albumin, total cholesterol, LDL-C, HDL-C, TG, fasting glucose, 25(OH)D, hs-CRP, eGFR, spot urine ACR, LVMI, and LVEF. The distributions of achieved femur neck BMD are also shown (bars). Abbreviations: BMD, bone mineral density; HR, hazard ratio.

To compare the cumulative incidence of all-cause mortality by BMD at femur neck, a Kaplan–Meier curve was analyzed (Figure 2), which depicted a significantly increased number of all-cause death events in subjects with osteopenia or osteoporosis during the follow-up period compared with subjects with normal BMD. To address an independent association between low BMD at femur neck and increased risk of all-cause mortality, Cox regression models were analyzed (Table 2). Osteopenia was associated with an increased risk of all-cause mortality only before adjustment with covariates (unadjusted HR 1.798, 95% CI 1.256 to 2.575) but not in the fully adjusted model (adjusted HR 1.115, 95% CI 0.745 to 1.661). In contrast, osteoporosis was significantly associated with an increased risk of all-cause mortality both in the crude (unadjusted HR 4.191, 95% CI 2.520 to 6.971) and fully adjusted (adjusted HR 2.963, 95% CI 1.655 to 5.307) models. To examine a possible nonlinear association between BMD and the risk of all-cause mortality, a smoothing curve fitting model was analyzed (Figure 3), which demonstrated a clear inverse correlation between BMD and the risk of all-cause mortality even after adjustment with confounding factors. Taken together, low BMD in patients with nondialysis CKD was associated with an increased risk of all-cause mortality.

### 3.3. Sensitivity Analyses

To test whether the association was still valid with BMD of the other anatomical sites and lumbar spine, the study participants were recategorized by BMD T-scores at total hip (Supplementary Tables S2 and S3) or lumbar spine (Supplementary Tables S4 and S5). Kaplan–Meier curve analysis demonstrated that, compared with subjects with normal BMD, the cumulative incidence of all-cause mortality was increased in subjects with osteopenia and osteoporosis defined by low BMD T-scores at total hip (Supplementary Figure S1) or lumbar spine (Supplementary Figure S2). Cox regression analysis revealed that osteoporosis, but not osteopenia, defined by BMD T-scores at total hip (adjusted HR 4.734, 95% CI 1.938 to 11.566) or lumbar spine (adjusted HR 2.456, 95% CI 1.353 to 4.459) was significantly associated with an increased risk of all-cause mortality (Supplementary Table S6). Smoothing curve fitting models visualized that BMD T-scores both in total hip (Supplementary Figure S3) and lumbar spine (Supplementary Figure S4) were inversely correlated with the risk of all-cause mortality, although the extremely high BMD T-score of lumbar spine was unexpectedly associated with a slight increase in the risk of all-cause mortality. After excluding subjects with CKD stage 1, osteoporosis defined by BMD T-scores of femur neck (adjusted HR 3.307, 95% CI 1.833 to 5.965), total hip (adjusted HR 4.839, 95% CI 2.013 to 11.634), or lumbar spine (adjusted HR 2.603, 95% CI 1.424 to 4.760) were still significantly associated with an increased risk of all-cause mortality (Supplementary Table S7). Even after excluding subjects with CKD stage 5, osteoporosis defined by BMD T-scores of femur neck (adjusted HR 3.221, 95% CI 1.719 to 6.035), total hip (adjusted HR 4.213, 95% CI 1.657 to 10.712), or lumbar spine (adjusted HR 2.585, 95% CI 1.334 to 5.010) were robustly and significantly associated with an increased risk of all-cause mortality (Supplementary Table S8). After replacing the missing values by multiple imputation, osteoporosis defined by BMD T-scores of femur neck (adjusted HR 3.590, 95% CI 2.142 to 6.018), total hip (adjusted HR 4.978, 95% CI 2.377 to 10.426), or lumbar spine (adjusted HR 2.987, 95% CI 1.745 to 5.112) remained significantly associated with an increased risk of all-cause mortality (Supplementary Table S9). After including total calcium, phosphorus, and iPTH as covariables in the regression models, osteoporosis defined by BMD T-scores of femur neck (adjusted HR 2.072, 95% CI 1.040 to 4.127), total hip (adjusted HR 5.260, 95% CI 1.986 to 13.933), or lumbar spine (adjusted HR 1.389, 95% CI 1.572 to 5.593) were significantly associated with an increased risk of all-cause mortality (Supplementary Table S10).

### 3.4. Subgroup Analysis

To examine whether the association of low BMD with the risk of all-cause mortality is modified by clinical contexts, we conducted a series of subgroup analyses (Table 3), though the association between low BMD and the risk of all-cause mortality was not altered by age (<60 versus (vs.) ≥60 years), gender (male vs. female), BMI (<23 kg/m^2^ vs. ≥23 kg/m^2^), eGFR (≥45 mL/min./1.73 m^2^ vs. <45 mL/min./1.73 m^2^), or spot urine ACR (<300 mg/g vs. ≥300 mg/g).

## 4. Discussion

In the present study, we found that low BMD is associated with an increased risk of all-cause mortality in patients with nondialysis CKD. All BMD T-scores measured at femur neck, total hip, and lumbar spine were inversely associated with the risk of mortality. The association was not modified by certain clinical contexts, such as age, gender, BMI, eGFR, and albuminuria.

The association between low BMD and all-cause mortality has been well described in the general population [10] and in patients with ESRD [17,20] but not in patients with nondialysis CKD. To our best knowledge, this is the first report to validate the association among patients with CKD who have not reached ESRD yet. This emphasizes that the routine measurement of BMD by DXA may confer an additional benefit beyond the prediction of fracture risk in this population.

A possible explanation for the association could be that patients with low BMD are at higher risk of fracture events. It is now generally accepted that low BMD predicts the risk of fracture not only in the general population but also in patients with nondialysis CKD or ESRD [9]. Moreover, the risk of fracture is higher in patients with CKD than in the general population [25], while the fracture is clearly associated with an increased risk of death [26]. Therefore, a potential contribution of increased fracture risk to all-cause mortality among subjects with CKD and low BMD seems convincing. 

On the other hand, the association between low BMD and all-cause mortality may be dependent on accelerated vascular calcification. The association between defective mineralization of bone (i.e., low BMD) and ectopic vascular calcification has been repeatedly reported [27,28,29] and is referred to as the ‘calcification paradox’ [30]. In addition, uremic milieu, such as hyperphosphatemia, is likely to promote vascular calcification, as a previous study reported that exposure to high phosphate media induces osteogenic differentiation of vascular smooth muscle cells in vitro [31]. Indeed, a recent cohort study of patients with nondialysis CKD proved that low BMD is associated with the progression of coronary artery calcification [15]. Hence, the risk of cardiovascular events in relation to low BMD may also explain the association between low BMD and all-cause mortality in this population.

Several factors differentially affect the site of bone loss, as bone loss in postmenopausal women is more prominent in trabecular-rich sites (e.g., vertebrae) [32,33], whereas CKD progression mostly results in cortical bone loss rather than trabecular bone loss [34,35]. Femur neck is a cortical-rich site, and low BMD at this site is expected to more precisely predict adverse clinical outcomes among patients with impaired renal function [36], which is best delineated by a report that low BMD at femur neck, but not other anatomical sites, is associated with all-cause mortality in patients with ESRD [20]. In contrast, our finding is that BMD T-scores measured at femur neck as well as at total hip and lumbar spine were inversely associated with the risk of mortality among patients with nondialysis CKD. This might imply that cortical bone loss in patients with nondialysis CKD is not as severe as in patients with ESRD.

Calcification of abdominal aorta, which is prevalent in patients with ESRD, may inappropriately overestimate BMD in lumbar spine [36]. This may also be a cause for the low predictability of lumbar spine BMD in the mortality among patients with ESRD [20,36]. Although low BMD at lumbar spine was significantly associated with an increased risk of all-cause mortality in the current study, the restricted cubic spline curve showed that extremely high BMD T-scores of lumbar spine were associated with a slight increase in risk (Figure S4), which could be possibly attributed to the artifact of aortic calcification in the measurement of BMD by central DXA. 

There are some limitations in the current study. First, due to the observational nature of the study, we were not able to confirm the causal relationship between low BMD and the risk of all-cause mortality. However, similar findings from the general population [10] and from patients with ESRD [17,18,19,20] strongly suggest that low BMD should increase the risk of all-cause mortality in patients with nondialysis CKD. Second, as we did not include the causes of death in the analysis, we could not determine whether low BMD was associated not only with traumatic mortality but also with nontraumatic mortality. Yet, recent studies reported that low BMD in patients with CKD increased the risk of incident ESRD [16] or cardiovascular events [15], strongly suggesting that the mortality observed in patients with low BMD and CKD may not be entirely due to traumatic injury. Third, because all of the study participants in the present study were limited to Korean residents in South Korea, the extrapolation of the results of the study to the other populations requires precaution. Nevertheless, similar results among patients with ESRD have been reported in studies conducted in other countries [17,18,19,20].

## 5. Conclusions

In conclusion, we report that low BMD is associated with an increased risk of all-cause mortality in patients with nondialysis CKD.

## Figures and Tables

**Table 1:** Baseline characteristics of study participants by femur neck BMD.

		Femur Neck BMD		
	Normal	Osteopenia	Osteoporosis	*p-*Value
Follow-up duration (year)	7.281 ± 2.805	7.011 ± 2.853	6.173 ± 3.421	0.003
Age (year)	54.312 ± 12.399	50.890 ± 12.004	56.989 ± 9.978	<0.001
Male	931 (62.0)	297 (60.5)	55 (58.5)	0.690
CCI				<0.001
0–3	1126 (75.0)	315 (64.2)	52 (55.3)	
4–5	357 (23.8)	166 (33.8)	38 (40.4)	
6–7	17 (1.1)	10 (2.0)	4 (4.3)	
≥8	1 (0.1)	0 (0.0)	0 (0.0)	
Primary renal disease				<0.001
DM	341 (22.7)	145 (29.5)	36 (38.3)	
HTN	279 (18.6)	114 (23.2)	17 (18.1)	
GN	483 (32.2)	157 (32.0)	24 (25.5)	
TID	7 (0.5)	5 (1.0)	1 (1.1)	
PKD	297 (19.8)	38 (7.7)	4 (4.3)	
Others	93 (6.2)	32 (6.5)	12 (12.8)	
Smoking status				<0.001
Nonsmoker	746 (49.7)	287 (58.5)	75 (79.8)	
Exsmoker	274 (18.3)	58 (11.8)	5 (5.3)	
Current smoker	480 (32.0)	146 (29.7)	14 (14.9)	
Medication				
ACEIs/ARBs	1285 (85.6)	422 (85.9)	78 (83.0)	0.752
Diuretics	430 (28.6)	184 (37.5)	41 (43.6)	<0.001
Anti-HTN drugs ≥3	409 (27.2)	168 (34.2)	31 (33.0)	0.009
Statins	727 (48.4)	297 (60.5)	61 (64.9)	<0.001
BMI (kg/m^2^)	24.9 ± 3.4	23.9 ± 3.3	23.4 ± 3.4	<0.001
Waist circumference (cm)	88.1 ± 9.8	85.9 ± 9.5	84.1 ± 10.2	<0.001
SBP (mmHg)	128.1 ± 16.1	127.2 ± 15.5	127.1 ± 19.6	0.526
DBP (mmHg)	77.7 ± 11.1	75.6 ± 10.8	73.5 ± 11.6	<0.001
Laboratory findings				
Hemoglobin (g/dL)	13.19 ± 2.00	12.15 ± 1.88	11.482 ± 1.56	<0.001
Albumin (g/dL)	4.20 ± 0.42	4.14 ± 0.42	4.07 ± 0.46	0.001
Total cholesterol (mg/dL)	175 ± 38	171 ± 40	169 ± 42	0.165
HDL-C (mg/dL)	49 ± 15	50 ± 16	48 ± 16	0.614
LDL-C (mg/dL)	98 ± 32	93 ± 31	91 ± 33	0.004
TG (mg/dL)	161 ± 104	151 ± 89	145 ± 75	0.040
Fasting glucose (mg/dL)	112 ± 41	107 ± 28	116 ± 52	0.026
25(OH)D (ng/mL)	17.70 ± 7.18	18.36 ± 9.66	16.50 ± 9.06	0.156
Total calcium (mg/dL)	9.15 ± 0.52	9.07 ± 0.57	9.00 ± 0.55	0.001
Phosphorus (mg/dL)	3.62 ± 0.67	3.85 ± 0.69	3.91 ± 0.68	<0.001
iPTH (pg/mL)	46 (29, 75)	60 (40, 106)	83 (51, 136)	<0.001
hs-CRP (mg/dL)	0.60 (0.20, 1.70)	0.67 (0.23, 1.60)	0.60 (0.30, 1.65)	0.761
Spot urine ACR (mg/g)	301.78 (58.89, 2213.74)	478.45 (127.23, 1183.61)	501.49 (172.33, 4876.12)	0.001
Creatinine (mg/dL)	1.69 ± 1.08	2.09 ± 1.27	2.34 ± 1.25	<0.001
eGFR (mL/min./1.73 m^2^)	55.24 ± 30.91	40.21 ± 25.06	32.60 ± 24.13	<0.001
CKD stages				<0.001
Stage 1	301 (20.1)	38 (7.7)	3 (3.2)	
Stage 2	325 (21.7)	65 (13.2)	6 (6.4)	
Stage 3a	263 (17.5)	67 (13.6)	12 (12.8)	
Stage 3b	296 (19.7)	127 (25.9)	16 (17.0)	
Stage 4	259 (17.3)	143 (29.1)	40 (42.6)	
Stage 5	57 (3.8)	51 (10.4)	17 (18.1)	

Values for categorical variables are given as number (percentage); values for continuous variables as mean ± standard deviation or median [interquartile range]. Abbreviations: 25(OH)D, 25-hydroxyvitamin D; ACEIs, angiotensin-converting enzyme inhibitors; ACR, albumin-to-creatinine ratio; ARBs, angiotensin receptor blockers; BMD, bone mineral density; BMI, body mass index; CCI, Charlson comorbidity index; CKD, chronic kidney disease; DBP, diastolic blood pressure; DM, diabetes mellitus; eGFR, estimated glomerular filtration rate; GN, glomerulonephritis; HDL-C, high-density lipoprotein cholesterol; hs-CRP, high-sensitivity C-reactive protein; HTN, hypertension; iPTH, intact parathyroid hormone; LDL-C, low-density lipoprotein cholesterol; PKD, polycystic kidney disease; SBP, systolic blood pressure; TGs, triglycerides; TID, tubulointerstitial disease.

**Table 2:** HRs for all-cause mortality by femur neck BMD.

	BMD	Events, *n* (%)	Model 1		Model 2		Model 3		Model 4	
			HR (95%CI)	*p-*Value	HR (95%CI)	*p-*Value	HR (95%CI)	*p-*Value	HR (95%CI)	*p-*Value
Femur neck BMD	Normal	100 (6.7)	Reference		Reference		Reference		Reference	
	Osteopenia	52 (10.6)	1.798	0.001	1.104	0.589	1.090	0.657	1.115	0.534
			(1.256, 2.575)		(0.771, 1.582)		(0.745, 1.596)		(0.748, 1.661)	
	Osteoporosis	23 (24.5)	4.191	<0.001	3.273	<0.001	2.922	<0.001	2.963	<0.001
			(2.520, 6.971)		(1.998, 5.361)		(1.707, 5.001)		(1.655, 5.307)	

Model 1, unadjusted model. Model 2, model 1 + adjusted for age, sex, Charlson comorbidity index, primary renal disease, smoking status, medication (ACEIs/ARBs, diuretics, number of anti-HTN drugs, statins), BMI, and SBP. Model 3, model 2 + adjusted for hemoglobin, albumin, total cholesterol, LDL-C, HDL-C, TG, fasting glucose, 25(OH)D, and hs-CRP. Model 4, model 3 + adjusted for eGFR, spot urine ACR, LVMI, and LVEF. Abbreviations: BMD, bone mineral density; CI, confidence interval; HR, hazard ratio.

**Table 3:** HRs for the primary outcome by non-HDL-C level in various subgroups.

	Femur Neck BMD	Events, *n* (%)	Unadjusted HR (95%CI)	*p* for Interaction	Adjusted HR (95%CI)	*p* for Interaction
Age <60 years	Normal	62 (6.6)	Reference	0.888	Reference	0.321
	Osteopenia	40 (11.0)	1.747 (1.174, 2.600)		1.397 (0.872, 2.236)	
	Osteoporosis	12 (25.0)	4.577 (2.466, 8.495)		1.933 (0.808, 4.625)	
Age ≥60 years	Normal	38 (6.7)	Reference		Reference	
	Osteopenia	12 (9.5)	1.466 (0.766, 2.807)		1.706 (0.727, 4.006)	
	Osteoporosis	11 (23.9)	4.309 (2.202, 8.433)		9.676 (3.723, 25.151)	
Male	Normal	56 (6.0)	Reference	0.845	Reference	0.798
	Osteopenia	26 (8.8)	1.517 (0.953, 2.415)		1.399 (0.789, 2.479)	
	Osteoporosis	11 (20.0)	4.295 (2.250, 8.200)		3.744 (1.635, 8.573)	
Female	Normal	44 (7.7)	Reference		Reference	
	Osteopenia	26 (13.4)	1.845 (1.136, 2.996)		1.408 (0.785, 2.526)	
	Osteoporosis	12 (30.8)	4.407 (2.327, 8.346)		3.433 (1.435, 8.215)	
BMI < 23 kg/m^2^	Normal	27 (6.1)	Reference	0.770	Reference	0.226
	Osteopenia	19 (9.6)	1.737 (0.965, 3.124)		1.556 (0.736, 3.287)	
	Osteoporosis	9 (20.5)	3.928 (1.846, 8.355)		2.253 (0.691, 7.350)	
BMI ≥ 23 kg/m^2^	Normal	73 (7.3)	Reference		Reference	
	Osteopenia	32 (11.1)	1.604 (1.059, 2.431)		1.364 (0.824, 2.257)	
	Osteoporosis	14 (28.0)	5.127 (2.893, 9.087)		4.326 (2.145, 8.721)	
eGFR ≥ 45 mL/min./1.73 m^2^	Normal	31 (3.7)	Reference	0.434	Reference	0.156
	Osteopenia	6 (3.8)	1.079 (0.450, 2.586)		0.656 (0.209, 2.061)	
	Osteoporosis	3 (18.8)	6.306 (1.927, 20.636)		11.773 (2.768, 50.073)	
eGFR < 45 mL/min./1.73 m^2^	Normal	69 (10.5)	Reference		Reference	
	Osteopenia	46 (13.7)	1.348 (0.928, 1.958)		1.780 (1.142, 2.774)	
	Osteoporosis	20 (25.6)	2.838 (1.725, 4.670)		3.775 (1.957, 7.283)	
Spot urine ACR < 300 mg/g	Normal	33 (4.6)	Reference	0.072	Reference	0.248
	Osteopenia	17 (8.9)	2.129 (1.186, 3.822)		1.276 (0.628, 2.593)	
	Osteoporosis	10 (29.4)	8.526 (4.198, 17.315)		4.538 (1.853, 11.113)	
Spot urine ACR ≥ 300 mg/g	Normal	63 (8.5)	Reference		Reference	
	Osteopenia	34 (11.9)	1.423 (0.938, 2.160)		1.603 (0.966, 2.658)	
	Osteoporosis	12 (21.4)	2.831 (1.527, 5.249)		2.135 (0.851, 5.356)	

Adjusted HR of non-HDL-C as a continuous variable for composite CV event is depicted. The model was adjusted for age, sex, Charlson comorbidity index, primary renal disease, smoking status, medication (ACEIs/ARBs, diuretics, number of anti-HTN drugs, statins), BMI, SBP, hemoglobin, albumin, total cholesterol, LDL-C, HDL-C, TG, fasting glucose, 25(OH)D, hs-CRP, eGFR, spot urine ACR, LVMI, and LVEF. Abbreviations: BMD, bone mineral density; CI, confidence interval; eGFR, estimated glomerular filtration rate; HR, hazard ratio.

## Data Availability

The raw data supporting the conclusions of this article will be made available by the authors without undue reservation.

## Supplementary Materials

The following are available online at https://www.mdpi.com/article/10.3390/jcm12051850/s1, Figure S1: Kaplan–Meier survival curve for cumulative incidence of all-cause mortality by total hip BMD, Figure S2: Kaplan–Meier survival curve for cumulative incidence of all-cause mortality by lumbar spine BMD, Figure S3: restricted cubic spline of total hip BMD on all-cause mortality, Figure S4: restricted cubic spline of lumbar spine BMD on all-cause mortality, Table S1: summary of echocardiographic findings of study participants by femur neck BMD, Table S2: baseline characteristics of study participants by total hip BMD, Table S3: summary of echocardiographic findings of study participants by total hip BMD, Table S4: baseline characteristics of study participants by lumbar spine BMD, Table S5: summary of echocardiographic findings of study participants by lumbar spine BMD, Table S6: HRs for all-cause mortality by total hip and lumbar spine BMD, Table S7: HRs for all-cause mortality by BMD after excluding subjects at CKD stage 1, Table S8: HRs for all-cause mortality by BMD after excluding subjects at CKD stage 5, Table S9: HRs for all-cause mortality by BMD using multiple imputation, Table S10: HRs for all-cause mortality by BMD in a model including serum calcium, phosphorus, and iPTH as covariables.
