# Supplementary material for: Bone Mineral Density and All-Cause Mortality in Patients with Nondialysis Chronic Kidney Disease: Results from KNOW-CKD Study
Source: J Clin Med. 2023 Feb 25;12(5):1850. doi: 10.3390/jcm12051850 (PMC10003778; doi:10.3390/jcm12051850)
Supplement: Supplementary file 1 [file jcm-12-01850-s001.zip › jcm-2198669-supplementary.pdf]

Journal of
Clinical Medicine

SUPPLEMENTARY MATERIALS

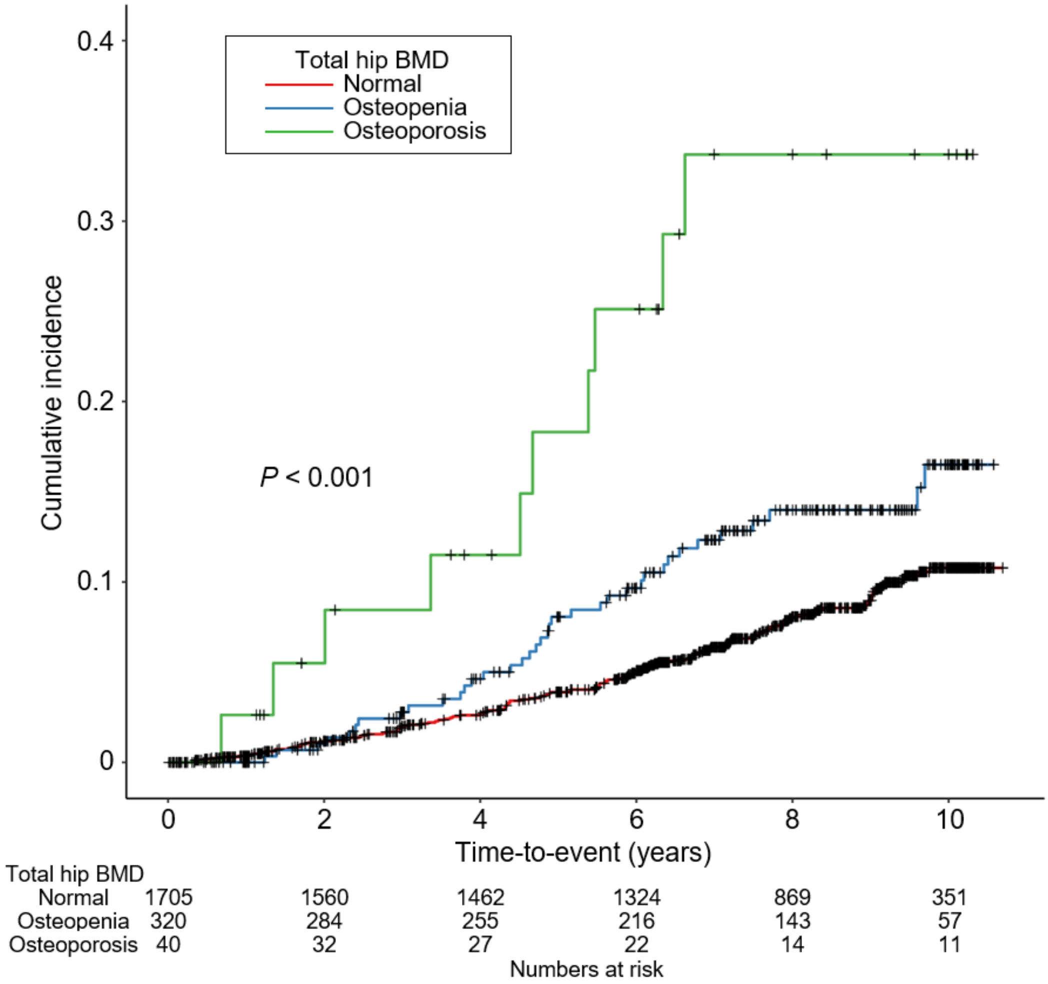

Figure S1. Kaplan-Meier survival curve for cumulative incidence of all-cause mortality by total hip BMD. *P* value by Log-rank test. Abbreviations: BMD, bone mineral density.

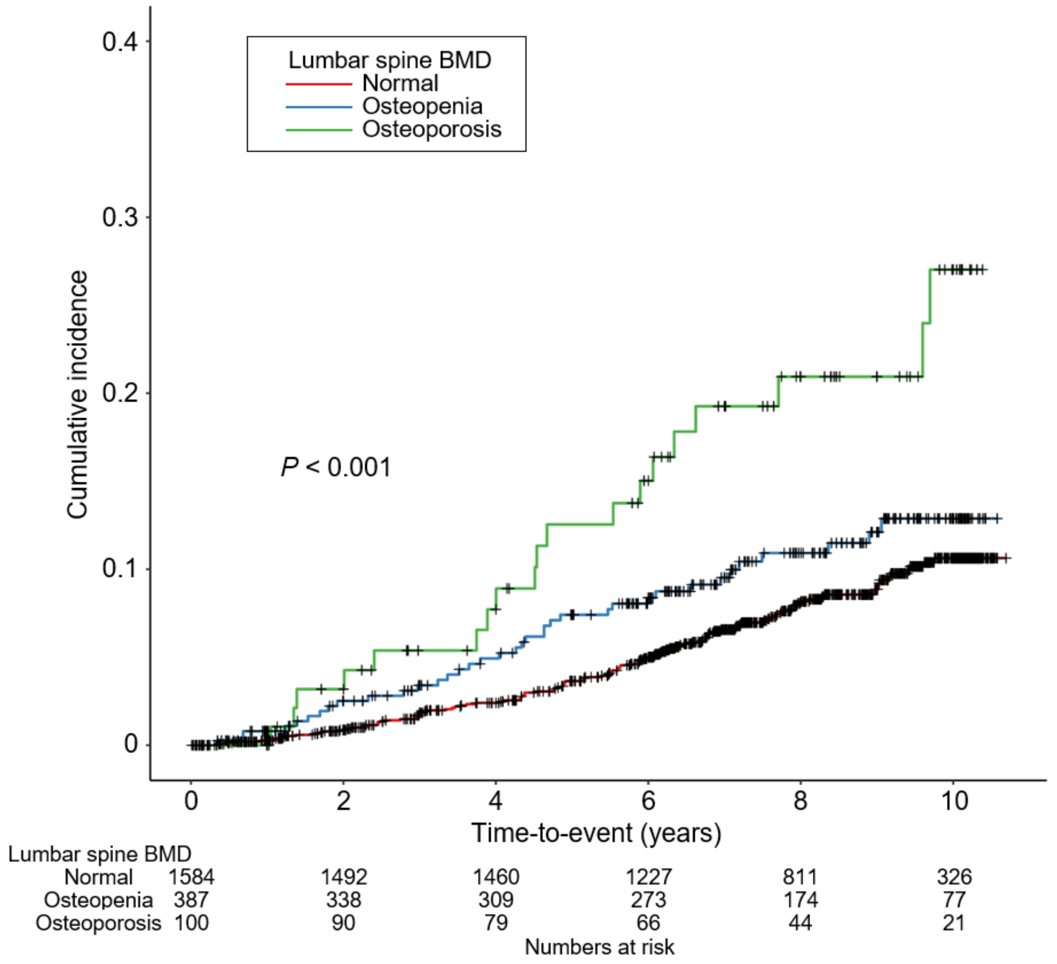

Figure S2. Kaplan-Meier survival curve for cumulative incidence of all-cause mortality by lumbar spine BMD. *P* value by Log-rank test. Abbreviations: BMD, bone mineral density.

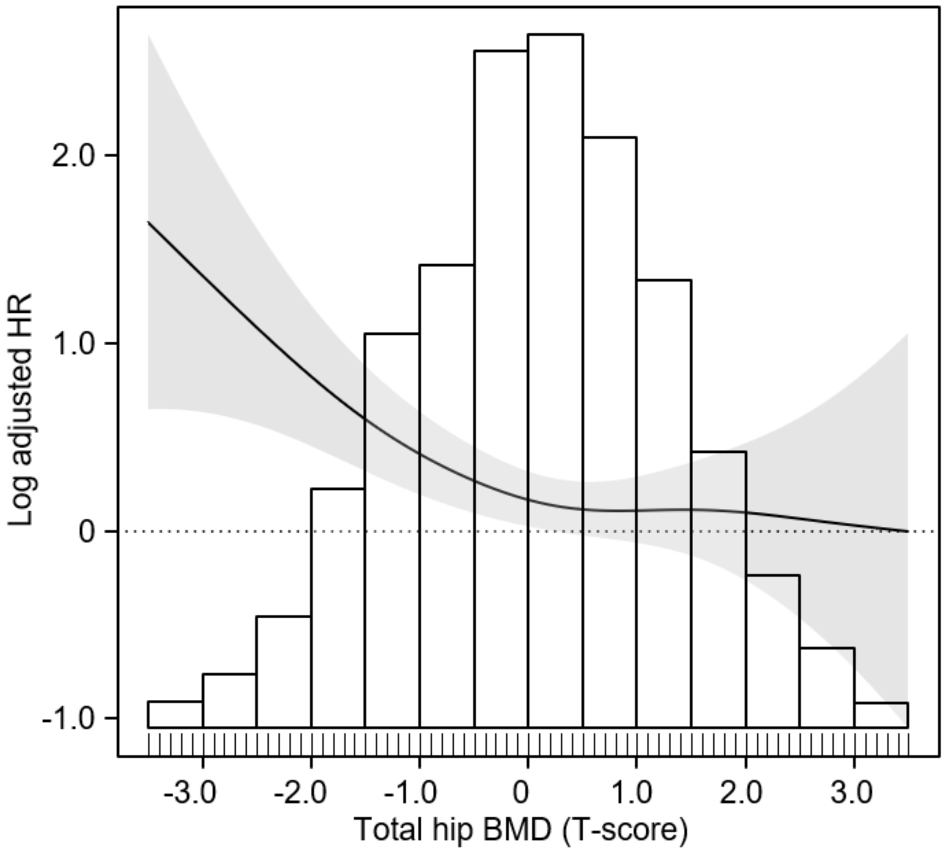

Figure S3. Restricted cubic spline of total hip BMD on all-cause mortality. Adjusted HR of non-HDL-C as a continuous variable for composite CV event is depicted. The model was adjusted for age and sex, Charlson comorbidity index, primary renal disease, smoking status, medication (ACEIs/ARBs, diuretics, number of anti-HTN drugs, statins), BMI, SBP, hemoglobin, albumin, total cholesterol, LDL-C, HDL-C, TG, fasting glucose, 25(OH)D, hs-CRP, eGFR, spot urine ACR, LVMI, and LVEF. Abbreviations: BMD, bone mineral density; HR, hazard ratio.

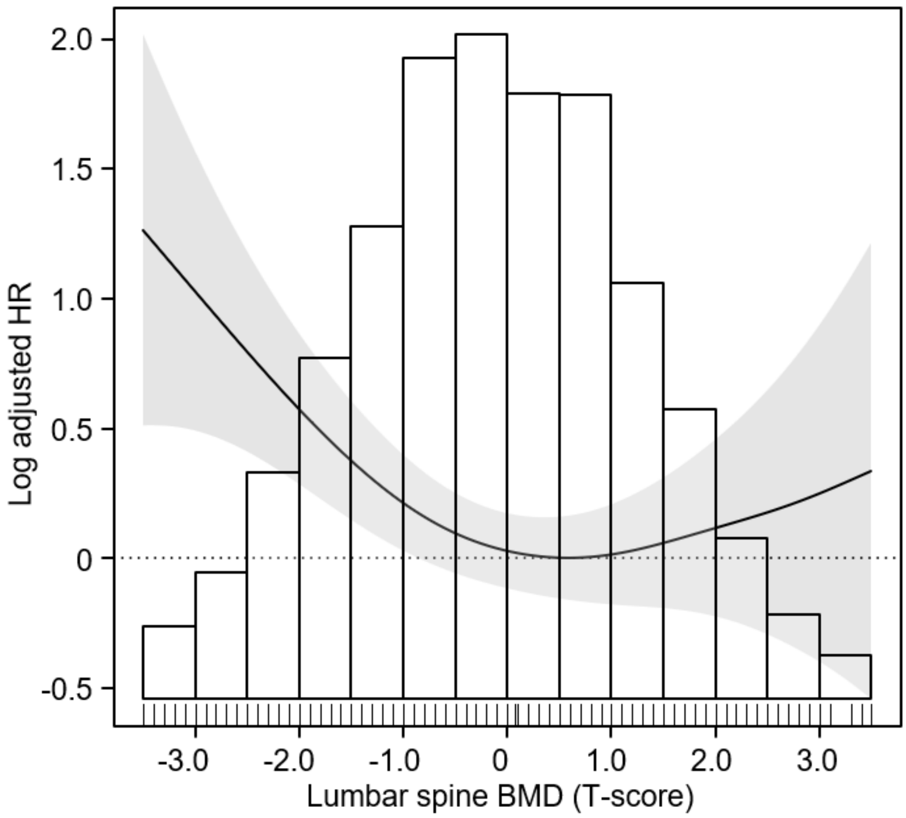

Figure S4. Restricted cubic spline of lumbar spine BMD on all-cause mortality. Adjusted HR of non-HDL-C as a continuous variable for composite CV event is depicted. The model was adjusted for age and sex, Charlson comorbidity index, primary renal disease, smoking status, medication (ACEIs/ARBs, diuretics, number of anti-HTN drugs, statins), BMI, SBP, hemoglobin, albumin, total cholesterol, LDL-C, HDL-C, TG, fasting glucose, 25(OH)D, hs-CRP, eGFR, spot urine ACR, LVMI, and LVEF. Abbreviations: BMD, bone mineral density; HR, hazard ratio.

Table S1. Summary of echocardiographic findings of study participants by femur neck BMD.

	Femur neck BMD			
	Normal	Osteopenia	Osteoporosis	*P* value
LVMI (g/m^2)	92.888 ± 25.610	95.964 ± 23.617	95.662 ± 22.421	0.045
E/e'	9.534 ± 3.480	10.871 ± 4.345	11.821 ± 6.119	<0.001
LVEF (%)	63.836 ± 6.097	64.617 ± 6.767	64.949 ± 6.258	0.029
LAD (mm)	37.806 ± 5.685	37.896 ± 6.326	36.845 ± 6.043	0.301
RWMA	48 (3.2)	14 (2.9)	1 (1.1)	0.435
Valve calcification	75 (5.0)	87 (17.8)	20 (21.5)	< 0.001
PWT (mm)	9.276 ± 1.626	9.257 ± 1.500	9.306 ± 1.441	0.948
IVWT (mm)	9.335 ± 1.746	9.414 ± 1.635	9.421 ± 1.793	0.624
LVEDD (mm)	48.965 ± 4.379	48.182 ± 4.560	46.877 ± 4.820	<0.001
LVESD (mm)	30.637 ± 4.187	29.983 ± 4.368	29.088 ± 4.246	<0.001

Values for categorical variables are given as number (percentage); values for continuous variables, as mean ± standard deviation or median [interquartile range]. Abbreviations: BMD, bone mineral density; E/e′, ratio of the early transmitral blood flow velocity to early diastolic velocity of the mitral annulus; IVWT, interventricular wall thickness; LAD, left atrium diameter; LVEDD, left ventricular end-diastolic diameter; LVEF, left ventricular ejection fraction; LVESD, left ventricular end-systolic diameter; LVMI, left ventricular mass index; PWT, posterior wall thickness; RMWA, regional wall motion abnormality.

Table S2. Baseline characteristics of study participants by total hip BMD.

	Total hip BMD			
	Normal	Osteopenia	Osteoporosis	*P* value
Follow-up duration (year)	7.272 ± 2.793	6.815 ± 2.993	5.894 ± 3.461	0.003
Age (year)	53.673 ± 12.487	53.141 ± 11.322	55.050 ± 11.893	0.559
Male	1041 (61.1)	204 (63.7)	24 (60.0)	0.650
CCI				0.069
0 – 3	1243 (72.9)	208 (65.0)	28 (70.0)	
4 – 5	438 (25.7)	105 (32.8)	11 (27.5)	
6 – 7	23 (1.3)	7 (2.2)	1 (2.5)	
≥ 8	1 (0.1)	0 (0.0)	0 (0.0)	
Primary renal disease				< 0.001
DM	410 (24.1)	97 (30.3)	12 (30.0)	
HTN	334 (19.6)	68 (21.2)	5 (12.5)	
GN	546 (32.0)	102 (31.9)	13 (32.5)	
TID	9 (0.5)	3 (0.9)	1 (2.5)	
PKD	306 (18.0)	27 (8.4)	2 (5.0)	
Others	99 (5.8)	23 (7.2)	7 (17.5)	
Smoking status				< 0.001
Non-smoker	843 (49.5)	221 (69.1)	36 (90.0)	
Ex-smoker	301 (17.7)	32 (10.0)	1 (2.5)	
Current smoker	560 (32.9)	67 (20.9)	3 (7.5)	
Medication				
ACEIs/ARBs	1469 (86.2)	268 (83.8)	32 (80.0)	0.311
Diuretics	516 (30.3)	120 (37.5)	14 (35.0)	0.034
Anti-HTN drugs ≥ 3	494 (29.0)	101 (31.6)	8 (20.0)	0.280
Statins	880 (51.6)	171 (53.4)	25 (62.5)	0.346
BMI (kg/m^2)	24.839 ± 3.377	23.350 ± 3.310	23.249 ± 4.098	< 0.001
Waist circumference (cm)	88.024 ± 9.726	84.413 ± 9.839	84.861 ± 9.521	< 0.001
SBP (mmHg)	128.035 ± 15.946	126.756 ± 16.622	126.900 ± 21.008	0.434
DBP (mmHg)	77.437 ± 10.958	74.972 ± 11.463	75.975 ± 12.550	0.002
Laboratory findings				
Hemoglobin (g/dL)	13.082 ± 2.013	11.873 ± 1.730	11.503 ± 1.588	< 0.001
Albumin (g/dL)	4.195 ± 0.419	4.105 ± 0.458	4.062 ± 0.412	0.002
Total cholesterol (mg/dL)	173.745 ± 38.241	174.242 ± 41.856	172.077 ± 43.766	0.952
HDL-C (mg/dL)	48.920 ± 15.039	50.250 ± 17.080	52.316 ± 17.775	0.243
LDL-C (mg/dL)	96.884 ± 31.489	95.701 ± 32.815	88.928 ± 30.468	0.249
TG (mg/dL)	159.909 ± 102.000	151.087 ± 87.840	134.564 ± 76.458	0.053
Fasting glucose (mg/dL)	111.115 ± 40.024	108.450 ± 30.882	117.763 ± 66.987	0.332
25(OH)D	17.826 ± 7.448	17.649 ± 9.758	17.384 ± 11.520	0.931
hs-CRP (mg/dL)	0.600 [0.200, 1.600]	0.600 [0.218, 1.800]	0.500 [0.225, 2.350]	0.497
Spot urine ACR (mg/g)	329.778 [68.731, 1033.559]	439.741 [133.036, 3008.859]	502.513 [180.423, 1520.302]	0.030
Creatinine (mg/dL)	1.759 ± 1.145	2.085 ± 1.205	1.988 ± 0.992	< 0.001
eGFR (mL/min./1.73m^2)	53.038 ± 30.524	40.033 ± 27.189	37.138 ± 24.644	< 0.001
CKD stages				< 0.001
Stage 1	310 (18.2)	29 (9.1)	1 (2.5)	
Stage 2	351 (20.6)	36 (11.2)	5 (12.5)	
Stage 3a	295 (17.3)	40 (12.5)	4 (10.0)	
Stage 3b	349 (20.5)	70 (21.9)	13 (32.5)	
Stage 4	320 (18.8)	105 (32.8)	13 (32.5)	
Stage 5	80 (4.7)	40 (12.5)	4 (10.0)	

Values for categorical variables are given as number (percentage); values for continuous variables, as mean ± standard deviation or median [interquartile range]. Abbreviations: 25(OH)D, 25-hydroxyvitamin D; ACEIs, angiotensin converting enzyme inhibitors; ACR, albumin-to-creatinine ratio; ARBs, angiotensin receptor blockers; BMD, bone mineral density; BMI, body mass index; CCI, Charlson comorbidity index; CKD, chronic kidney disease; DBP, diastolic blood pressure; DM, diabetes mellitus; eGFR, estimated

glomerular filtration rate; GN, glomerulonephritis; HDL-C, high density lipoprotein cholesterol; hs-CRP, high-sensitivity C-reactive protein; HTN, hypertension; LDL-C, low density lipoprotein cholesterol; PKD, polycystic kidney disease; SBP, systolic blood pressure; TG, triglycerides; TID, tubulointerstitial disease.

Table S3. Summary of echocardiographic findings of study participants by total hip BMD.

	Total hip BMD			
	Normal	Osteopenia	Osteoporosis	*P* value
LVMI (g/m^2)	93.294 ± 25.488	95.077 ± 23.273	96.879 ± 19.498	0.298
E/e'	9.757 ± 3.740	10.904 ± 4.673	10.836 ± 3.513	< 0.001
LVEF (%)	64.026 ± 6.304	64.510 ± 6.306	64.032 ± 5.381	0.459
LAD (mm)	37.888 ± 5.656	37.326 ± 6.825	36.444 ± 5.876	0.139
RWMA	51 (3.0)	10 (3.1)	0 (0.0)	0.192
Valve calcification	128 (7.5)	44 (13.8)	5 (12.5)	0.001
PWT (mm)	9.273 ± 1.607	9.215 ± 1.476	9.313 ± 1.679	0.805
IVWT (mm)	9.351 ± 1.736	9.329 ± 1.630	9.724 ± 1.886	0.449
LVEDD (mm)	48.980 ± 4.373	47.290 ± 4.574	46.815 ± 5.293	< 0.001
LVESD (mm)	30.643 ± 4.218	29.368 ± 4.168	28.900 ± 4.282	< 0.001

Values for categorical variables are given as number (percentage); values for continuous variables, as mean ± standard deviation or median [interquartile range]. Abbreviations: BMD, bone mineral density; E/e', ratio of the early transmitral blood flow velocity to early diastolic velocity of the mitral annulus; IVWT, interventricular wall thickness; LAD, left atrium diameter; LVEDD, left ventricular end-diastolic diameter; LVEF, left ventricular ejection fraction; LVESD, left ventricular end-systolic diameter; LVMI, left ventricular mass index; PWT, posterior wall thickness; RMWA, regional wall motion abnormality.

Table S4. Baseline characteristics of study participants by lumbar spine BMD.

	Lumbar spine BMD			
	Normal	Osteopenia	Osteoporosis	*P* value
Follow-up duration (year)	7.287 ± 2.775	6.823 ± 3.062	6.771 ± 3.068	0.010
Age (year)	53.483 ± 12.520	53.832 ± 11.850	55.250 ± 11.017	0.292
Male	966 (61.0)	247 (63.8)	63 (63.0)	0.564
CCI				0.917
0 – 3	1130 (71.3)	280 (72.4)	76 (76.0)	
4 – 5	428 (27.0)	103 (26.6)	23 (23.0)	
6 – 7	25 (1.6)	4 (1.0)	1 (1.0)	
≥ 8	1 (0.1)	0 (0.0)	0 (0.0)	
Primary renal disease				0.375
DM	406 (25.6)	91 (23.5)	18 (18.0)	
HTN	317 (20.0)	70 (18.1)	20 (20.0)	
GN	504 (31.8)	125 (32.3)	31 (31.0)	
TID	10 (0.6)	2 (0.5)	1 (1.0)	
PKD	249 (15.7)	66 (17.1)	24 (24.0)	
Others	97 (6.1)	33 (8.5)	6 (6.0)	
Smoking status				< 0.001
Non-smoker	798 (50.4)	231 (59.7)	73 (73.0)	
Ex-smoker	273 (17.2)	57 (14.7)	3 (3.0)	
Current smoker	512 (32.3)	99 (25.6)	24 (24.0)	
Medication				
ACEIs/ARBs	1353 (85.4)	332 (85.8)	87 (87.0)	0.900
Diuretics	501 (31.6)	112 (28.9)	33 (33.0)	0.547
Anti-HTN drugs ≥ 3	467 (29.5)	108 (27.9)	30 (30.0)	0.817
Statins	814 (51.4)	215 (55.6)	44 (44.0)	0.094
BMI (kg/m^2)	24.929 ± 3.401	23.495 ± 3.177	22.968 ± 3.449	< 0.001
Waist circumference (cm)	88.349 ± 9.857	84.751 ± 9.097	82.797 ± 9.191	< 0.001
SBP (mmHg)	127.901 ± 16.219	127.674 ± 15.915	126.220 ± 15.108	0.559
DBP (mmHg)	77.046 ± 11.170	77.052 ± 10.866	75.620 ± 10.195	0.399
Laboratory findings				
Hemoglobin (g/dL)	13.069 ± 2.029	12.377 ± 1.853	11.651 ± 1.798	< 0.001
Albumin (g/dL)	4.187 ± 0.429	4.165 ± 0.414	4.123 ± 0.429	0.263
Total cholesterol (mg/dL)	173.659 ± 39.042	171.922 ± 38.436	178.550 ± 39.286	0.316
HDL-C (mg/dL)	48.895 ± 15.294	49.811 ± 15.458	51.264 ± 17.417	0.276
LDL-C (mg/dL)	96.455 ± 31.943	95.715 ± 31.569	97.218 ± 27.593	0.875
TG (mg/dL)	162.039 ± 103.645	143.228 ± 80.313	145.357 ± 83.650	< 0.001
Fasting glucose (mg/dL)	112.062 ± 40.970	106.346 ± 31.591	106.071 ± 37.699	0.007
25(OH)D (ng/dL)	17.810 ± 7.828	18.100 ± 8.344	17.151 ± 7.848	0.569
hs-CRP (mg/dL)	0.600 [0.250, 1.623]	0.600 [0.200, 1.600]	0.600 [0.300, 2.100]	0.374
Spot urine ACR (mg/g)	346.304 [71.370, 1053.503]	347.704 [95.962, 1042.392]	396.539 [96.460, 1067.667]	0.902
Creatinine (mg/dL)	1.770 ± 1.137	1.923 ± 1.177	2.010 ± 1.176	0.016
eGFR (mL/min./1.73m^2)	52.378 ± 30.273	46.416 ± 29.721	40.965 ± 27.782	< 0.001
CKD stages				< 0.001
Stage 1	279 (17.6)	53 (13.7)	8 (8.0)	
Stage 2	324 (20.5)	60 (15.5)	11 (11.0)	
Stage 3a	270 (17.0)	52 (13.4)	17 (17.0)	
Stage 3b	322 (20.3)	92 (23.8)	23 (23.0)	
Stage 4	312 (19.7)	98 (25.3)	26 (26.0)	
Stage 5	77 (4.9)	32 (8.3)	15 (15.0)	

Values for categorical variables are given as number (percentage); values for continuous variables, as mean ± standard deviation or median [interquartile range]. Abbreviations: 25(OH)D, 25-hydroxyvitamin D; ACEIs, angiotensin converting enzyme inhibitors; ACR, albumin-to-creatinine ratio; ARBs, angiotensin receptor blockers; BMD, bone mineral density; BMI, body mass index; CCI, Charlson comorbidity index; CKD, chronic kidney disease; DBP, diastolic blood pressure; DM, diabetes mellitus; eGFR, estimated

glomerular filtration rate; GN, glomerulonephritis; HDL-C, high density lipoprotein cholesterol; hs-CRP, high-sensitivity C-reactive protein; HTN, hypertension; LDL-C, low density lipoprotein cholesterol; PKD, polycystic kidney disease; SBP, systolic blood pressure; TG, triglycerides; TID, tubulointerstitial disease.

Table S5. Summary of echocardiographic findings of study participants by lumbar spine BMD.

	Lumbar spine BMD			
	Normal	Osteopenia	Osteoporosis	*P* value
LVMI (g/m^2)	93.652 ± 25.832	94.175 ± 23.513	92.840 ± 18.479	0.836
E/e’	9.770 ± 3.693	10.558 ± 4.719	10.363 ± 3.669	0.005
LVEF (%)	63.892 ± 6.166	64.567 ± 6.845	65.075 ± 5.864	0.046
LAD (mm)	37.953 ± 5.813	37.073 ± 5.812	37.904 ± 6.809	0.031
RWMA	49 (3.1)	12 (3.1)	2 (2.0)	0.872
Valve calcification	117 (7.4)	56 (14.5)	9 (9.0)	< 0.001
PWT (mm)	9.331 ± 1.625	9.088 ± 1.525	9.041 ± 1.123	0.003
IVWT (mm)	9.398 ± 1.763	9.237 ± 1.635	9.101 ± 1.319	0.040
LVEDD (mm)	48.916 ± 4.469	48.188 ± 4.464	46.863 ± 4.179	< 0.001
LVESD (mm)	30.586 ± 4.243	29.992 ± 4.358	29.225 ± 3.897	0.001

Values for categorical variables are given as number (percentage); values for continuous variables, as mean ± standard deviation or median [interquartile range]. Abbreviations: BMD, bone mineral density; E/e′, ratio of the early transmitral blood flow velocity to early diastolic velocity of the mitral annulus; IVWT, interventricular wall thickness; LAD, left atrium diameter; LVEDD, left ventricular end-diastolic diameter; LVEF, left ventricular ejection fraction; LVESD, left ventricular end-systolic diameter; LVMI, left ventricular mass index; PWT, posterior wall thickness; RMWA, regional wall motion abnormality.

Table S6. HRs for the all-cause mortality by total hip and lumbar spine BMD.

	BMD	Events, n (%)	Model 1		Model 2		Model 3		Model 4	
			HR (95%CIs)	*P* value	HR (95%CIs)	*P* value	HR (95%CIs)	*P* value	HR (95%CIs)	*P* value
Total hip	Normal	127 (7.4)	Reference		Reference		Reference		Reference	
	Osteopenia	37 (11.6)	1.698 (1.140, 2.530)	0.009	1.299 (0.876, 1.924)	0.193	1.188 (0.781, 1.807)	0.421	1.126 (0.731, 1.733)	0.590
	Osteoporosis	10 (25.0)	3.814 (1.775, 8.195)	< 0.001	5.199 (2.591, 10.432)	< 0.001	6.277 (2.845, 13.846)	< 0.001	4.734 (1.938, 11.566)	< 0.001
Lumbar spine	Normal	117 (7.4)	Reference		Reference		Reference		Reference	
	Osteopenia	37 (9.6)	1.404 (0.938, 2.101)	0.099	1.351 (0.919, 1.987)	0.126	1.245 (0.822, 1.887)	0.301	1.286 (0.835, 1.980)	0.253
	Osteoporosis	19 (19.0)	2.814 (1.660, 4.770)	< 0.001	2.622 (1.557, 4.416)	< 0.001	2.341 (1.335, 4.104)	0.003	2.456 (1.353, 4.459)	0.003

Model 1, unadjusted model. Model 2, model 1 + adjusted for age, sex, Charlson comorbidity index, primary renal disease, smoking status, medication (ACEIs/ARBs, diuretics, number of anti-HTN drugs, statins), BMI, and SBP. Model 3, model 2 + adjusted for hemoglobin, albumin, total cholesterol, LDL-C, HDL-C, TG, fasting glucose, 25(OH)D, and hs-CRP. Model 4, model 3 + adjusted for eGFR, spot urine ACR, LVMI, and LVEF. Abbreviations: BMD, bone mineral density; CI, confidence interval; HR, hazard ratio.

Table S7. HRs for the all-cause mortality by BMD after excluding the subjects at CKD stage 1.

	BMD	Events, n (%)	Model 1		Model 2		Model 3		Model 4	
			HR (95%CIs)	*P* value	HR (95%CIs)	*P* value	HR (95%CIs)	*P* value	HR (95%CIs)	*P* value
Femur neck	Normal	97 (8.1)	Reference		Reference		Reference		Reference	
	Osteopenia	52 (11.5)	1.662 (1.131, 2.328)	0.009	1.354 (0.949, 1.933)	0.095	1.384 (0.946, 2.024)	0.094	1.400 (0.940, 2.086)	0.098
	Osteoporosis	22 (24.2)	3.369 (1.998, 5.681)	< 0.001	3.806 (2.312, 6.263)	< 0.001	3.256 (1.890, 5.609)	< 0.001	3.307 (1.833, 5.965)	< 0.001
Total hip	Normal	124 (8.9)	Reference		Reference		Reference		Reference	
	Osteopenia	36 (12.4)	1.520 (1.014, 2.278)	0.043	1.562 (1.055, 2.311)	0.026	1.389 (0.911, 2.119)	0.127	1.290 (0.834, 1.995)	0.252
	Osteoporosis	10 (25.6)	3.352 (1.559, 7.207)	0.002	5.628 (2.810, 11.271)	< 0.001	5.746 (2.613, 12.363)	< 0.001	4.839 (2.013, 11.634)	< 0.001
Lumbar spine	Normal	115 (8.8)	Reference		Reference		Reference		Reference	
	Osteopenia	36 (10.8)	1.351 (0.897, 2.033)	0.150	1.352 (0.917, 1.994)	0.128	1.379 (0.908, 2.094)	0.132	1.467 (0.954, 2.256)	0.081
	Osteoporosis	18 (19.6)	2.420 (1.405, 4.168)	0.001	2.776 (1.645, 4.685)	< 0.001	2.510 (1.422, 4.431)	0.001	2.603 (1.424, 4.760)	0.002

Model 1, unadjusted model. Model 2, model 1 + adjusted for age, sex, Charlson comorbidity index, primary renal disease, smoking status, medication (ACEIs/ARBs, diuretics, number of anti-HTN drugs, statins), BMI, and SBP. Model 3, model 2 + adjusted for hemoglobin, albumin, total cholesterol, LDL-C, HDL-C, TG, fasting glucose, 25(OH)D, and hs-CRP. Model 4, model 3 + adjusted for eGFR, spot urine ACR, LVMI, and LVEF. Abbreviations: BMD, bone mineral density; CI, confidence interval; HR, hazard ratio.

Table S8. HRs for the all-cause mortality by BMD after excluding the subjects at CKD stage 5.

	BMD	Events, n (%)	Model 1		Model 2		Model 3		Model 4	
			HR (95%CIs)	*P* value	HR (95%CIs)	*P* value	HR (95%CIs)	*P* value	HR (95%CIs)	*P* value
Femur neck	Normal	93 (6.4)	Reference		Reference		Reference		Reference	
	Osteopenia	43 (9.8)	1.709 (1.155, 2.527)	0.007	1.239 (0.846, 1.815)	0.271	1.244 (0.827, 1.871)	0.295	1.252 (0.817, 1.919)	0.303
	Osteoporosis	17 (22.1)	4.102 (2.322, 7.248)	< 0.001	3.475 (2.000, 6.035)	< 0.001	3.166 (1.738, 5.768)	< 0.001	3.221 (1.719, 6.035)	< 0.001
Total hip	Normal	115 (7.1)	Reference		Reference		Reference		Reference	
	Osteopenia	29 (10.4)	1.606 (1.028, 2.510)	0.038	1.443 (0.941, 2.215)	0.093	1.314 (0.828, 2.085)	0.246	1.187 (0.734, 1.917)	0.485
	Osteoporosis	8 (22.2)	3.797 (1.664, 8.664)	0.002	4.898 (2.273, 10.552)	< 0.001	4.591 (1.903, 11.079)	< 0.001	4.213 (1.657, 10.712)	0.003
Lumbar spine	Normal	108 (7.2)	Reference		Reference		Reference		Reference	
	Osteopenia	30 (8.5)	1.215 (0.775, 1.904)	0.395	1.297 (0.852, 1.973)	0.225	1.272 (0.809, 2.000)	0.298	1.405 (0.879, 2.247)	0.155
	Osteoporosis	14 (16.5)	2.584 (1.416, 4.717)	0.002	2.825 (1.582, 5.045)	< 0.001	2.393 (1.259, 4.546)	0.008	2.585 (1.334, 5.010)	0.005

Model 1, unadjusted model. Model 2, model 1 + adjusted for age, sex, Charlson comorbidity index, primary renal disease, smoking status, medication (ACEIs/ARBs, diuretics, number of anti-HTN drugs, statins), BMI, and SBP. Model 3, model 2 + adjusted for hemoglobin, albumin, total cholesterol, LDL-C, HDL-C, TG, fasting glucose, 25(OH)D, and hs-CRP. Model 4, model 3 + adjusted for eGFR, spot urine ACR, LVMI, and LVEF. Abbreviations: BMD, bone mineral density; CI, confidence interval; HR, hazard ratio.

Table S9. HRs for the all-cause mortality by BMD using multiple imputation.

	BMD	Model 1		Model 2		Model 3		Model 4	
		HR (95%CIs)	*P* value	HR (95%CIs)	*P* value	HR (95%CIs)	*P* value	HR (95%CIs)	*P* value
Femur neck	Normal	Reference		Reference		Reference		Reference	
	Osteopenia	1.669 (1.194, 2.334)	0.003	1.422 (1.002, 2.018)	0.051	1.333 (0.930, 1.912)	0.120	1.275 (0.886, 1.836)	0.192
	Osteoporosis	4.463 (2.836, 7.023)	< 0.001	4.215 (2.592, 6.857)	< 0.001	3.663 (2.230, 6.017)	< 0.001	3.590 (2.142, 6.018)	< 0.001
Total hip	Normal	Reference		Reference		Reference		Reference	
	Osteopenia	1.660 (1.151, 2.394)	0.007	1.646 (1.123, 2.414)	0.012	1.414 (0.955, 2.093)	0.085	1.312 (0.885, 1.944)	0.178
	Osteoporosis	4.375 (2.288, 8.364)	< 0.001	6.190 (3.106, 12.336)	< 0.001	5.890 (2.830, 12.257)	< 0.001	4.978 (2.377, 10.426)	< 0.001
Lumbar spine	Normal	Reference		Reference		Reference		Reference	
	Osteopenia	1.385 (0.958, 2.004)	0.085	1.500 (1.028, 2.188)	0.037	1.510 (1.027, 2.221)	0.038	Reference	0.041
	Osteoporosis	2.004 (1.712, 4.513)	< 0.001	3.185 (1.912, 5.306)	< 0.001	2.879 (1.700, 4.875)	< 0.001	2.987 (1.745, 5.112)	< 0.001

Model 1, unadjusted model. Model 2, model 1 + adjusted for age, sex, Charlson comorbidity index, primary renal disease, smoking status, medication (ACEIs/ARBs, diuretics, number of anti-HTN drugs, statins), BMI, and SBP. Model 3, model 2 + adjusted for hemoglobin, albumin, total cholesterol, LDL-C, HDL-C, TG, fasting glucose, 25(OH)D, and hs-CRP. Model 4, model 3 + adjusted for eGFR, spot urine ACR, LVMI, and LVEF. Abbreviations: BMD, bone mineral density; CI, confidence interval; HR, hazard ratio.

Table S10. HRs for the all-cause mortality by BMD in a model including serum calcium, phosphorus, and iPTH as covariables.

	BMD	Model 1		Model 2		Model 3		Model 4	
		HR (95%CIs)	*P* value	HR (95%CIs)	*P* value	HR (95%CIs)	*P* value	HR (95%CIs)	*P* value
Femur neck	Normal	Reference		Reference		Reference		Reference	
	Osteopenia	1.448 (0.955, 3.068)	0.007	1.149 (0.742, 1.780)	0.533	1.135 (0.724, 1.779)	0.581	1.039 (0.660, 1.635)	0.870
	Osteoporosis	3.068 (1.699, 5.540)	< 0.001	2.771 (1.464, 5.243)	0.002	2.402 (1.229, 4.695)	0.010	2.072 (1.040, 4.127)	0.038
Total hip	Normal	Reference		Reference		Reference		Reference	
	Osteopenia	1.459 (0.914, 2.329)	0.113	1.366 (0.838, 2.226)	0.212	1.143 (0.690, 1.891)	0.604	1.079 (0.651, 1.788)	0.767
	Osteoporosis	3.983 (1.843, 8.608)	< 0.001	6.187 (2.627, 14.572)	< 0.001	5.608 (2.119, 14.845)	< 0.001	5.260 (1.986, 13.933)	0.001
Lumbar spine	Normal	Reference		Reference		Reference		Reference	
	Osteopenia	1.292 (0.805, 2.072)	0.288	1.436 (0.885, 2.330)	0.143	1.393 (0.828, 243)	0.212	1.389 (0.827, 2.332)	0.214
	Osteoporosis	3.037 (1.743, 5.275)	0.002	3.357 (1.846, 6.106)	< 0.001	3.220 (1.724, 6.013)	0.008	2.965 (1.572, 5.593)	0.001

Model 1, unadjusted model. Model 2, model 1 + adjusted for age, sex, Charlson comorbidity index, primary renal disease, smoking status, medication (ACEIs/ARBs, diuretics, number of anti-HTN drugs, statins), BMI, and SBP. Model 3, model 2 + adjusted for hemoglobin, albumin, total cholesterol, LDL-C, HDL-C, TG, fasting glucose, calcium, phosphorus, iPTH, 25(OH)D, and hs-CRP. Model 4, model 3 + adjusted for eGFR, spot urine ACR, LVMI, and LVEF. Abbreviations: BMD, bone mineral density; CI, confidence interval; HR, hazard ratio.
